# Drone Secure Communication Protocol for Future Sensitive Applications in Military Zone

**DOI:** 10.3390/s21062057

**Published:** 2021-03-15

**Authors:** Yongho Ko, Jiyoon Kim, Daniel Gerbi Duguma, Philip Virgil Astillo, Ilsun You, Giovanni Pau

**Affiliations:** 1 Jeju Free International City Development Center, Jeju Island 63309, Korea; koyh0911@gmail.com; 2 Department of Information Security Engineering, Soonchunhyang University, Asan-si 31538, Korea; 74jykim@sch.ac.kr (J.K.); 20189512@sch.ac.kr (D.G.D.); 20189093@sch.ac.kr (P.V.A.); 3 Faculty of Engineering and Architecture, Kore University of Enna, 94100 Enna, Italy; giovanni.pau@unikore.it

**Keywords:** drone, security, formal verification, vulnerability, D2D, D2GCS, attacks

## Abstract

Unmanned Aerial Vehicle (UAV) plays a paramount role in various fields, such as military, aerospace, reconnaissance, agriculture, and many more. The development and implementation of these devices have become vital in terms of usability and reachability. Unfortunately, as they become widespread and their demand grows, they are becoming more and more vulnerable to several security attacks, including, but not limited to, jamming, information leakage, and spoofing. In order to cope with such attacks and security threats, a proper design of robust security protocols is indispensable. Although several pieces of research have been carried out with this regard, there are still research gaps, particularly concerning UAV-to-UAV secure communication, support for perfect forward secrecy, and provision of non-repudiation. Especially in a military scenario, it is essential to solve these gaps. In this paper, we studied the security prerequisites of the UAV communication protocol, specifically in the military setting. More importantly, a security protocol (with two sub-protocols), that serves in securing the communication between UAVs, and between a UAV and a Ground Control Station, is proposed. This protocol, apart from the common security requirements, achieves perfect forward secrecy and non-repudiation, which are essential to a secure military communication. The proposed protocol is formally and thoroughly verified by using the BAN-logic (Burrow-Abadi-Needham logic) and Scyther tool, followed by performance evaluation and implementation of the protocol on a real UAV. From the security and performance evaluation, it is indicated that the proposed protocol is superior compared to other related protocols while meeting confidentiality, integrity, mutual authentication, non-repudiation, perfect forward secrecy, perfect backward secrecy, response to DoS (Denial of Service) attacks, man-in-the-middle protection, and D2D (Drone-to-Drone) security.

## 1. Introduction

Unmanned Aerial Vehicles (UAVs) occupy an essential place in both military and civilian applications by playing a core role in criminal investigations, public safety organizations, transportation management facilities, and surveillance forces [1]. With the ability of dynamic mobility, quick reaction, and ease of deployment, UAVs offer new possibilities for different applications at a viable expense. In the last few years alone, networked UAVs have been a dominating area of research for different business organizations, such as Google, Facebook, Boeing, and Amazon.

High portability is one reason for interface twisting in UAV networking. Regardless of this, UAV-enabled systems support remote networks in the regions where physical interaction is troublesome or costly. It is apparent from the current research that UAVs are suitable for plenty of use cases, yet their deployments face a ton of difficulties and criticisms. Initially, the majority of the researches contend on the architectural structure of drone communication, which at present comes up short with regard to standard and unification. In addition, UAV-aided communication systems experience the ill effects of issues related to spectrum sharing [2].

Aside from these, UAV communications face specific issues identified with the architectural plan, deployment, and consistency, with broad and dependable networks alongside their security [3]. Normally, UAVs function remotely by receiving commands from the ground control stations. These command and control messages are transmitted over various channels with a variable transmission rate [4]. Since that information transmitted to/by UAVs is mainly over the air, and most of the information transferred are highly sensitive and critical [5], security is a primary concern in UAV communications. Therefore, the security of these channels in UAV systems is one of the essential requirements for robust communication between UAVs and/or between UAVs and the Ground Control Station (GCS).

The security vulnerabilities can prompt an assault on confidentiality, trustworthiness, validness, and accessibility of UAVs. Generally, cryptographic mechanisms accomplish message security and control signal assurance. Consequently, security concerns like unauthorized access, malicious control, unlawful association, or other malevolent attacks need to be mitigated effectively with limited or no consequences on the performance [6]. Recognizable proof of threats and their defense in UAV systems are critical issues to be dealt with by comprehensive and proficient methodologies.

Recently, a vulnerability has been discovered in the DJI UAVs that an attacker was able to exploit to gain user account information, which then led to UAV hijacking [6]. The attack is succeeded by intercepting users’ identification tokens by logging into the DJI forums and acting as a legitimate user. It is often the case that the administrator of the UAVs maintains information related to flight history, photographs taken during the flight, payment information, real-time access rights of UAV cameras, and location information. Accordingly, attacks on these devices, apart from other damages, may enable adversaries to leak such crucial information and violate the security and privacy of users. In general, UAVs lack suitable security mechanisms that protect them from various attacks while taking a good balance between performance and safety [7].

Such security issues, especially in a military setting, may bring devastating effects that put classified information in jeopardy. For instance, a session hijacking attack orchestrated in a military scenario enables an attacker to extract previously exchanged information and use it for different malicious activities. Additionally, communication among UAVs needs to be secured since they usually work in collaboration to achieve a specific objective, such as passing information in an ad-hoc manner. Another critical issue in the military environment, where sensitive information is transmitted and commands are triggered, is maintaining tractability. That is, any entity (UAV or GCS) should be accountable for its actions and should not be able to repudiate it. Consequently, the main aim of this paper is to design a secure UAV communication that is specially designed for military environments by which perfect forward secrecy is maintained, UAV-to-UAV (and UAV-to-GCS) communications are secured, and nonrepudiation is supported. The key contributions of this paper are listed as follows:

- A new protocol for UAV-to-UAV and UAV-to-GCS is proposed,
- A formal security analysis of the proposed protocol using BAN-logic and Scyther tool is carried out,
- A detailed comparative analysis based on security property and computational overhead between the proposed and existing protocols is given,
- The protocol is also implemented on a real UAV (powered by Raspberry Pi) and a Linux-based ground control station.
- The remainder of the paper is organized as follows: In Section 2, the state-of-the-art study of existing drone communication protocols is described. In Section 3 and Section 4, the proposed protocol is presented in detail, and a formal security analysis of the protocol is provided, respectively. In the final three sections, performance analysis, simulation results, and conclusion of the paper are provided, respectively.

## 2. Related Works

The development era of drones and communication technologies are tremendously growing, where the various specialist service providers and equipment sellers are bringing constant flow of new advancements, such as network accessibility [8], offloading strategies [9], path planning [10], and various applications [11,12,13]. These enhancements go hand in hand with industrial advancements, such as in References [14,15]. In particular to UAVs, the ongoing improvements emphasize the information rate and security, which includes secrecy, honesty, verification, and non-denial of transmitted information. UAVs have a risk of information leakage as they are remotely controlled or operated through predetermined missions in a resource-limited environment. With this regard, the cryptographic mechanisms are well-known solutions against the attacks in most UAV-based communications, which help to design robust security services. UAV communication, in general, involves the drones, network providers, ground control stations, and trusted third parties for authentications. Every entity plays a significant role in the entire communication process to safeguard the system from security breaches. To this end, various researchers have studied multiple security issues concerning UAVs, such as eavesdropping, network jamming, weak authentication, and mobility management issues [16,17].

Seo et al. [18] proposed a security solution for drone-enabled delivery service by utilizing White-Box Cryptography (WBC) as a product assurance instrument for UAV landing points and cryptographic resources, alongside Public Key Infrastructure (PKI) as a verification and non-repudiation technique. The principal goals of the proposed protocol are assurance of a secret key, information protection during capturing, and secure storage of information. The authors considered different security properties, such as confidentiality, integrity, non-repudiation, authentication, and software protection. Kriz and Gabrlik [19] proposed the UranusLink packet-oriented communication protocol with both non-reliable and reliable transfer mechanisms that allow secure connection and packet loss detection. The authors discussed various related issues such as security, low data throughput, ability to data loss detection, and low latency. Won et al. [20] proposed a secure communication protocol for drones and smart objects that depend on an efficient Certificateless Signcryption Tag Key Encapsulation Mechanism (eCLSC-TKEM). Islam et al. [21] presented a group key distribution protocol for FANETs (Flying Ad hoc NETworks), which relies on a group leader that discharges the base station for other operations. The authors considered different FANET requirements, such as node mobility and changes in the topology. Maxa et al. [22] provided a protected UAV ad hoc reactive routing protocol (SUAP; Secure Uav Ad hoc routing Protocol) that depends on public-key cryptography, hash chains, and geological lashes. It is utilized to ensure the route discovery component giving trustworthiness, verification, and non-repudiation services, which is the expansion of the SAODV (Secure Ad hoc On-demand Distance Vector) routing protocol.

Other related researches such as Blazy et al. [23] proposed UAV-GCS Secure Communication Protocol by using efficient cryptographic techniques to ensure the confidentiality of sensed data. The authors highlight various interesting requirements, such as forensic-resistant property of captured UAVs should not compromise the security of UAS (Unmanned Aerial System) or the freshness of keys, to name a few. In addition, Wang et al. [24] proposed a handover key management scheme for the LTE (Long-Term Evolution)-based UAV control system to stress on the robust and secure connection to direct and control the UAVs. The paper further discussed security prerequisites such as authentication, access control, confidentiality, integrity, and user plane traffic. A certificateless group authenticated key agreement (CL-GAKA) scheme for secure communication among untrusted parties is also proposed by Semal et al. [25]. The authors considered confidentiality, message integrity, and authenticity requirements in UAV communication along with UAV-to-UAV secure channel establishment, whereas UAV-to-Infrastructure communication, as well as the routing problem, are not discussed.

Another study that examined the security requirements of UAV communications is presented by He et al. [7]. The authors discussed specific attacks like GPS jamming, spoofing, and Wi-Fi attacks along with the countermeasures. Likewise, Kim et al. [26] proposed a mechanism to confirm deletion activities in the wake of eradicating information, regardless of whether control of a remotely conveyed UAV is lost. The authors utilized a countdown-based approach and a hash chain to validate the sender of the received messages to trigger the deletion activity, significantly after UAV communication was lost. In connection to this, the security and privacy concerns of the Internet of Drones (IoD) is studied by Wazid et al. [27]. The authors also proposed a centralized authentication and key agreement scheme. The authors cover various security requirements but lack emphasis on the forward and backward perfect secrecy and non-repudiation, which are the essential requirements in critical and sensitive drone-oriented missions.

## 3. The Proposed Protocol

This section describes a security protocol used for UAVs to communicate with monitoring UAVs and GCS. The protocol is mainly designed to serve in a military environment with two sub-protocols: SP-D2GS (Security Protocol for Drone-to-Ground Control Station) and SP-D2MD (Security Protocol for Drone-to-Monitoring Drone).

### 3.1. Preliminary

Apart from their widespread usage in many application areas, UAVs have been extensively used in military settings, especially for the purpose of surveillance, search and rescue, national intelligence programs, reconnaissance, etc. [28]. Clearly, such operations are sensitive by nature, due to the fact that they almost always involve national secrets. Consequently, if exchanged information between the UAVs and the ground station are disclosed, it may bring a lot of damages—from risking international relationships to serious conflicts and wars. Thus, it is important to design a scheme that enables communicating entities to establish a secure channel before exchanging any sensitive information. In this section, such a security protocol that is particularly designed to operate in a military environment is described.

**Figure 1:**
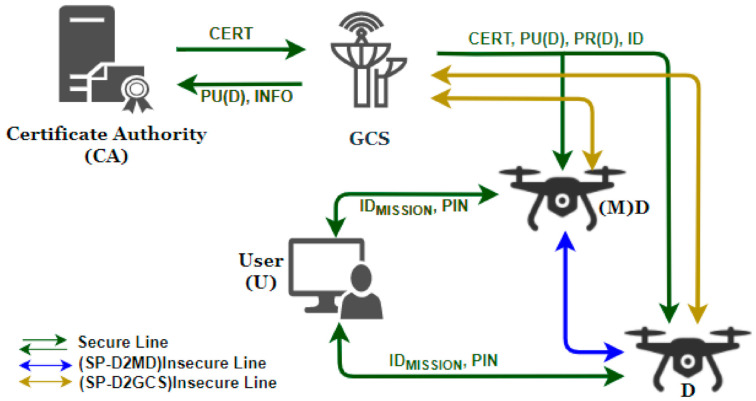
Execution flow of the proposed protocol.

The intended communication between the UAVs and the GCS can be arranged in a direct or hierarchical fashion. In the former case, each of the participating UAVs exchange information with the GCS independently. That is, the UAVs establish a secure channel with the GCS first, and send the collected data through a wireless channel. Such arrangements can be secured with the SP-D2GCS protocol (shown as the golden colored arrows in Figure 1). For the hierarchical organization, a dedicated monitoring drone is responsible to collect and transmit various data from each of the assigned UAVs to the GCS. The monitoring drone, hence, acts as a middleman that executes the SP-D2MD protocol (shown as the blue colored arrow in Figure 1) between the UAVs and itself, and then transmits the collected data to GCS by using the SP-D2GCS security protocol. The details of these sub-protocols will be described in Section 3.3 and Section 3.4.

Prior to the execution of the proposed protocol, however, the UAVs and the GCS need to be configured with the necessary information. First, the GCS generates the long-term private and public keys for each UAV. Then, it prepares a certificate request (CSR), based on their respective public keys and other information, and sends it to the Certificate Authority (CA). Next, it prepares unique identities (ID) for each of the participants. Once the key pairs, the certificates, and the IDs are ready, they will be securely delivered to each UAV, as shown by the green arrows in Figure 1. Furthermore, GCS and UAVs are assumed to be pre-configured with various cryptographic functions, such as digital signature algorithms (e.g., ECDSA; Elliptic Curve Digital Signature Algorithm), encryption and decryption function, cryptographic hash functions (e.g., HMAC; Hash-based Message Authentication Code), pseudo-random number generators (PRNG), etc. It is also assumed that the GCS and the UAVs are time-synchronized, and that the elliptic curve domain parameters (p, a, b, G, n, and h) are decided ahead of the communication, and are known by each of the communicating entities. Additionally, important information such as pre-shared keys (for instance PIN), IP address, type of UAV (monitoring or general drone), and operation ID (ID_MISSION_) are configured by the user before the UAVs start their mission.

### 3.2. Threat Model

In computing, a threat can be understood as any incident that has the potential to bring loss or harm to a system. Substantially, threats are events that aim at violating the confidentiality, integrity, and availability properties of a computing system. Such threats can happen due to different vulnerabilities, which are weaknesses in the system as a consequence of design flaws, configuration mistakes, security policy inaccuracies, to name a few. Consequently, anyone with malicious intent and technical capability can exploit these vulnerabilities to launch an attack, thereby realizing the threats. Attacks can be orchestrated by two classes of an adversary: insider or external. The former refers to malicious attacks, such as replay, falsification, and masquerading, repudiation, or obstructions [29]. These attacks are typically carried out by a foe with legitimate or authorized system access. The latter represents attacks committed on a system network or computer system mainly either by exploiting a vulnerability of the system or by social engineering. These are threat actors that attempt to exploit security exposures, and they are generally located outside the firewall.

More often than not, cryptographic protocols are intended to work in an open environment where adversaries are capable of accessing the ciphered information exchanged between communicating peers. Such security schemes are often modeled with the Dolev-Yao (DY) threat model [30]. This model assumes an insecure public channel (which makes the communicating entities untrustworthy) and powerful adversaries that are capable of obtaining messages passing through the network, initiate and receive a conversation to and from other participants, and able of impersonating other entities. Despite all these capacities of the attacker, there is off-limits information. Some of this information is guessing random numbers generated from sample space and deciphering a ciphertext, enciphering a plaintext, or getting the same HMAC value without the proper key. Consequently, the protocol proposed in this work is modeled using the DY threat model, and only GCS is assumed to be fully trusted.

The assumptions we took in designing this protocol are described as follows. It is assumed that the elliptic curve domain parameters (p, a, b, G, n, and h) are decided ahead of the communication and are known by each of the communicating entities. The GCS and all affiliated drones can obtain a timestamp value indicating the current time, and have time synchronization to verify the given timestamp value from the other party. The GCS and all its drones have public/private key pairs and certificates supporting Elliptic Curve Digital Signature Algorithm (ECDSA), GCS assigns IDs to the drones and monitoring drones, and the user plans the operation through the related application and selects the drones included in the operation by using ID_MISSION_ (the ID of the operation) and P (PIN number), which are provided before the execution of the protocol.

The proposed protocol is required to satisfy important security requirements to withstand various attacks. Some of the most important requirements are:

- Mutual Authentication: for secure communication among a drone, a monitoring drone, and a GCS, the communicating entities need to authenticate each other mutually.
- Strong Key Exchange: in order to assure the perfect forward secrecy of the protocol, a strong key exchange should be executed in a way that generated session keys cannot be recovered.
- Confidentiality: the information exchanged between the drones and between the drone and the GCS should be protected from being accessed by unauthorized parties.
- Integrity: it is critical to assure the authenticity of the information (that the information is not changed in between, and the source of information is genuine) exchanged between the communicating ends.
- Non-repudiation: one of the essential security requirements in such scenarios is to make sure that the action done by one party cannot be successfully denied without others knowing about it.
- Perfect Forward Secrecy: this property assures communicating parties that even if an adversary discloses a master key, old session keys will not be compromised.
- Perfect Backward Secrecy: this property assures the communicating entities that even if an adversary discloses a master key, future session keys will not be compromised.
- Protection against Denial of Service: legitimate users, such as legitimate drones, should not be denied service from a service provider, such as a GCS.
- Protection against MITM (Man-In-The-Middle) attack: the protocol prevents an attacker from secretly relaying messages between the communicating ends.

### 3.3. SP-D2GCS

**Figure 2:**
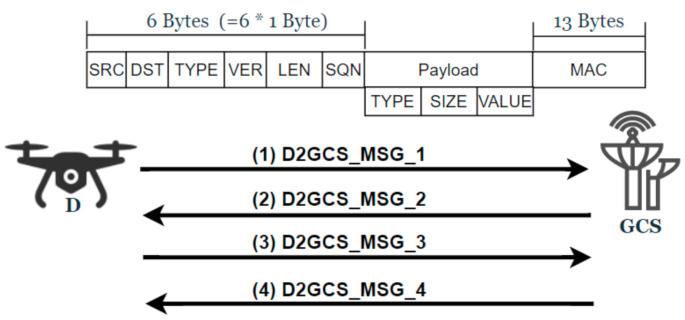
D2GCS communication and packet structure.

**Figure 3:**
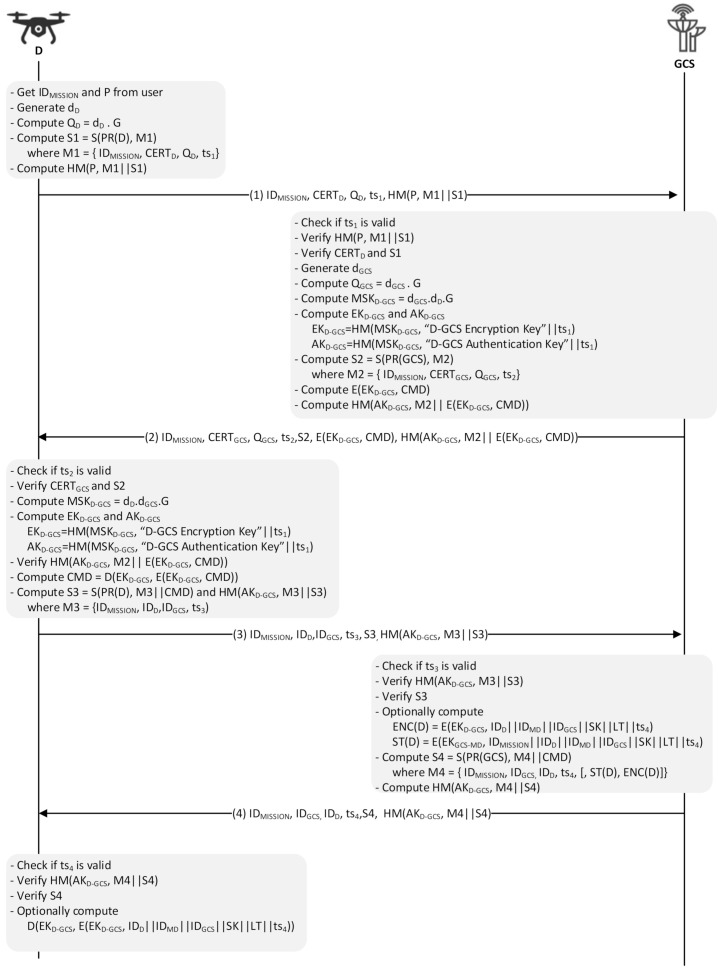
SP-D2GCS protocol.

The drones and GCS should establish a secure channel and mutually authenticate each other before exchanging any sensitive information. For this, a security protocol, SP-D2GCS (Security Protocol for Drone-to-Ground Control Station), is needed that operates between the drones and the GCS. In SP-D2GCS protocol, drones and a GCS securely communicate to exchange telemetry and status information (from the drone to GCS) and commands and controls (from GCS to the drones). The D2GCS protocol consists of four message exchanges and is also compatible with the defacto MAVLink packet structure [31]. The notations used in both sub protocols (SP-D2GCS and SP-D2MD) are described in Table 1. The communication and packet structure of the D2GCS protocol is shown in Figure 2, and the details of the proposed protocol are shown in Figure 3.

(1) The first thing that happens in the SP-D2GCS protocol is for D to get the operation ID (ID_MISSION_) and PIN (P) from the user. While doing so, or even before the actual protocol session starts, it can generate a random ECDH private key d_D_ ∈ {1… n − 1}, where n is the order of the group generated by G. It then calculates the ECDH public key Q_D_ = d_D_ • G. Now, D is ready to create a message M1, containing ID_MISSION_, its certificate (CERT_D_), the computed public key Q_D_, and the current timestamp ts_1_, which is accompanied with the signature S1 computed by the ECDSA private key PR(D). To allow GCS to prevent the resource exhaustion attacks caused by the expensive public key operation, an HMAC is computed over the message M1 and signature S1 using the PIN number, P. Finally, the message M1, with the signature S1 and the message digest, is sent to GCS.
(2) Upon receiving the message, GCS first checks its freshness by checking the included timestamp ts1. Once ts1 is in the acceptable threshold, it then computes HM(P, M1||S1), which is then compared with the received HMAC value. Note that doing two such verifications before the expensive public key operation, i.e., the S1 verification, helps to defend against resource exhaustion denial of service attacks. In a positive case, GCS checks the validity of the received certificate CERT_D_ and verifies the digital signature S1 by using the public key that belongs to CERT_D_. If the verification of S1 holds, GCS successfully authenticates D. Now, GCS uses the same procedure D followed to prepare the ECDH private key (d_GCS_) and public key (Q_GCS_ = d_GCS_ • G). It then computes the master session key MSK_D-GCS_ = d_GCS_•d_D_•G to produce the encryption and authentication keys. While the encryption key EK_D-GCS_ (=HM(MSKD-GCS, “D-GCS Encryption Key”||ts1)) is used to protect the confidentiality of the command CMD sent to D, the authentication key AKD-GCS (=HM(MS_KD-GCS_, “D-GCS Authentication Key”||ts_1_)) assures the authenticity and integrity of this command. GCS then arranges a message M2 (containing ID_MISSION_, CERT_GCS_, Q_GCS_, and ts_2_) and signs that message with its ECDSA private key PR(GCS), followed by encrypting the command CMD with the encryption key EK_D-GCS_ and computing HM(AK_D-GCS_, M2|| E(EK_D-GCS_, CMD)). Finally, GCS sends the message M2, the signature S2, the encrypted command, and the HMAC value to D.
(3) Once D gets the message, it verifies the timestamp ts_2_ and the digital signature S2 to authenticate GCS. Next, it generates the master session key MSK_D-GCS_, from which the encryption and authentication keys EK_D-GCS_ and AK_D-GCS_ are derived using the same procedure as shown in step (2). Afterward, D computes the HMAC value and verifies if it is the same as the one it received. In turn, it extracts the operation command CMD by decrypting the received cipher using EK_D-GCS_. To proceed with the next step, D further composes a message M3 (containing ID_MISSION_, ID_D_, ID_GCS_, and ts_3_), concatenates it with the deciphered CMD, and signs the result by computing S(PR(D), M3||CMD). It also calculates HM(AK_D-GCS_, M3||S3), which is, in turn, sent together with the message M3 and the digital signature S3 to GCS.
(4) Upon receipt of the message, GCS verifies the timestamp ts_3_ and the HMAC value before confirming the validity of the digital signature S3. If S3 is valid, GCS can be sure that D has successfully received the operation command CMD. S3 also plays an important role in fulfilling the non-repudiation property of the protocol by making sure that D cannot deny that it received the CMD. Similarly, GCS allows D to prove that it has sent an operation command CMD via the digital signature S4 (=S(PR(GCS), M4||CMD)). Besides, the HMAC value is calculated based on AK_D-GCS_ to counter the threat of the resource exhaustion attacks due to the public key operation. Note that in the SP-D2GCS protocol, GCS computes and transmits optional parameters that will be used for scenarios where drones communicate with their monitoring drone. In such scenarios, it prepares for a ticket that contains a session key SK and its lifetime LT along with the IDs of D and its monitoring drone MD. In more detail, GCS computes ENC(D) = E(E_KD-GCS_, ID_D_||ID_MD_||ID_GCS_||SK||LT||ts_4_) and ST(D) = E(EK_GCS-MD_, ID_MISSION_||ID_D_||ID_MD_||ID_GCS_||SK||LT||ts_4_) for D and MD, respectively. Finally, the GCS sends the message M4 (optionally including ENC(D) and ST(D)), the digital signature S4, and the HMAC value. The protocol is concluded after D validates the included ts_4_, HMAC value, and S4, respectively. Similar to S3, S4 supports non-repudiation. If ENC(D) and ST(D) are given, D recovers the session key SK by decrypting ENC(D) with EK_D-GCS_.

### 3.4. SP-D2MD

**Figure 4:**
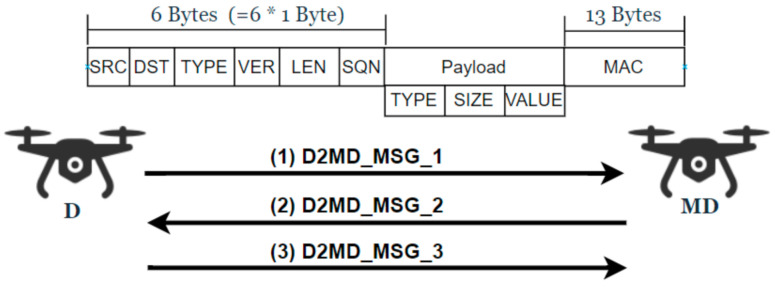
D2MD communication and packet structure.

**Figure 5:**
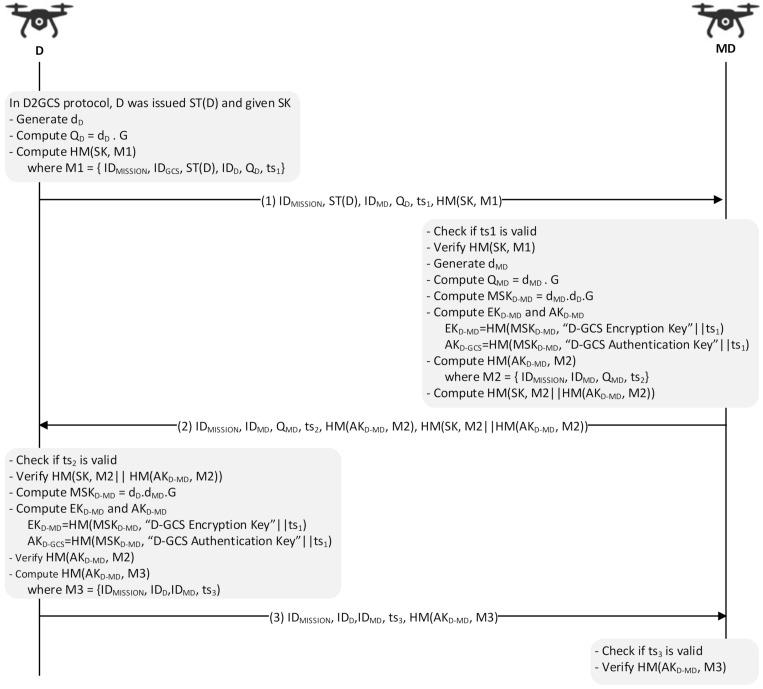
SP-D2MD protocol.

For cases where a dedicated monitoring drone is required to collect information from different general drones and pass this information to the ground station, a separate security protocol is required. Consequently, the SP-D2MD (Security Protocol for Drone-to-Monitoring Drone) protocol is used between a general drone D and a monitoring drone MD to perform mutual authentication and key exchange, thereby protecting their subsequent communications. Once all the information is collected by the MD, the MD uses the SP-D2GCS protocol to pass this information to GCS and receive different commands and controls from it. The communication and packet structure of this sub-protocol is shown in Figure 4, and the details are depicted in Figure 5.

(1) Note that during the D2GCS protocol session, D received the session key SK and the corresponding ticket ST(D) that allow itself to execute mutual authentication and key exchange with MD. To start this protocol, D first generates its ECDH public key pair d_D_ and Q_D_, before composing a message M1 containing ID_MISSION_, ID_GCS_, ST(D), ID_D_, Q_D_, and ts_1_. It, in turn, calculates HM(SK, M1), which is sent to MD along with M1.
(2) On receiving the message, MD verifies its freshness and decrypts ST(D) with EK_GCS-MD_ to extract SK, which is then used to verify the received HM(SK, M1). After that, it generates the ECDH public key pair d_MD_ and Q_MD_, computes a master session key MSK_D-MD_, and computes EK_D-MD_ and AK_D-MD_. Finally, D generates the two HMAC values, HM(AK_D-MD_, M2) and HM(SK, M2|| HM(AK_D-MD_, M2)), which are then sent to MD along with M2.
(3) After verifying the received ts_2_ and HM(SK, M2|| HM(AK_D-MD_, M2)), D computes MSK_D-MD_, EK_D-MD_, and AK_D-MD_. With AK_D-MD_, HM(AK_D-MD_, M2) is verified, followed by sending MD a message M3 (= ID_MISSION_, ID_D_, ID_MD_, ts_3_) with HM(AK_D-MD_, M3). Finally, MD concludes this protocol by verifying the included ts_3_ and HM(AK_D-MD_, M3). The positive result enables MD to confirm the valid key exchange.

## 4. Formal Security Analysis

This section puts forward the formal analysis of the proposed security protocols described in Section 3. The formal security analysis verifies whether the security protocol actually satisfies the targeted security requirements and services or not. In the past few years, the research on formal security analysis has been continuously conducted. In this paper, the proposed protocols are formally verified through modal-logic-based analysis, such as BAN Logic [32], and automation tool, such as Scyther [33].

### 4.1. Formal Verification with BAN-Logic

Named after its three authors, Burrows, Abadi, and Needham, BAN logic has become one of the most used verification methods to analyze security protocols formally. BAN-Logic consists of different notations and rules that are used for formal verification.

In general, formal verification through BAN-Logic is carried out in four steps: (1) idealization, (2) assumption, (3) goals, and (4) derivation. The analysis starts by idealizing the messages exchanged between the communicating parties by representing them into suitable format by which only encrypted (non-plaintext) messages are considered. Once the messages are put in this format, underlying assumptions regarding the original messages are made and formally expressed. Next, the goals are defined and expressed formally. Finally, the goals are derived by using the BAN-Logic rules, the assumptions, and the intermediate results. Here, ‘I’, ‘A’, ‘G’, and ‘D’ are used to denote idealizations, assumptions, goals, and derivations. Table 2 and Table 3 summarize the BAN-Logic notations and rules, respectively.

#### 4.1.1. SP-D2GCS

1. Idealization

The SP-D2GCS protocol is formulated into the following four idealizations.

(**I1**)  $$D\to GCS:\langle D_{MISSION},\;g^{x},\;ts_{1}\rangle {}_{p}$$
(**I2**)  $$GCS\to D:\langle ID_{MISSION},\;g^{y},\;ts_{2},CMD,GCS\overset{AK}{↔}D,GCS\overset{EK}{↔}D\rangle_{AK},{\left\{g^{y},\;ts_{2}\right\}}_{PU{\left(GCS\right)}^{-1}}$$
(**I3**)  $$D\to GCS:\;\langle ID_{MISSION},ID_{D},ID_{GCS},\;ts_{3},\;CMD,\;GCS\overset{AK}{↔}D,GCS\overset{EK}{↔}D\rangle_{AK}$$
(**I4**)  $$GCS\to D:\;\langle ID_{MISSION},ID_{GCS},ID_{D},\;ts_{4},\;CMD\rangle_{AK}$$

2. Assumptions

The assumptions taken in the process of verification are listed below. While the assumptions A1–A4, A6, and A10 are with respect to GCS, the rest are taken by D.

(**A1**)  $$\;GCS\;believes\;GCS\overset{P}{\Leftrightarrow }D$$
(**A2**)  $$\;GCS\;believes\;fresh\left(ts_{1}\right)$$
(**A3**)  $$\;GCS\;believes\;\overset{g^{Y}}{\to }GCS$$
(**A4**)  $$\;D\;believes\;\overset{PU\left(GCS\right)}{\to }GCS$$
(**A5**)  $$\;D\;believes\;fresh\left(ts_{2}\right)$$
(**A6**)  $$\;D\;believes\;\overset{g^{X}}{\to }D$$
(**A7**)  $$\;D\;believes\;fresh\left(ts_{1}\right)$$
(**A8**)  $$\;G\;believes\;fresh\left(ts_{3}\right)$$
(**A9**)  $$\;D\;believes\;fresh\left(ts_{4}\right)$$
(**A10**)  $$\;D\;believes\;GCS\;control\;D\overset{SK}{↔}MD$$

3. Goals

The goals that are expected to be met by the SP-D2GCS protocol are listed below. They primarily illustrate mutual authentication and secure key exchange between D and GCS.

(***G*1**)  $$\;GCS\;believes\;D\;believes\;ID_{MISSION}\;$$
(***G*2**)  $$\;GCS\;believes\;GCS\overset{AK}{↔}D$$
(***G*3**)  $$\;GCS\;believes\;GCS\overset{EK}{↔}D$$
(***G*4**)  $$\;D\;believes\;GCS\overset{AK}{↔}D\;$$
(***G*5**)  $$\;D\;believes\;GCS\overset{EK}{↔}D$$
(***G*6**)  $$\;D\;believes\;GCS\;believes\;ID_{MISSION}$$
(***G*7**)   D believes GCS believes CMD
(***G*8**)  $$\;D\;believes\;GCS\;believes\;GCS\overset{AK}{↔}D$$
(***G*9**)  $$\;D\;believes\;GCS\;believes\;GCS\overset{EK}{↔}D$$
(***G*10**)  $$\;GCS\;believes\;D\;believes\;ID_{D}$$
(***G*11**)   GCS believes D believes CMD
(***G*12**)  $$\;GCS\;believes\;D\;believes\;GCS\overset{AK}{↔}D$$
(***G*13**)  $$\;GCS\;believes\;D\;believes\;GCS\overset{EK}{↔}D$$
(***G*14**)  $$\;D\;believes\;GCS\;believes\;ID_{GCS}$$
(***G*15**)  $$\;D\;believes\;GCS\;believes\;D\overset{SK}{↔}MD$$
(***G*16**)  $$\;D\;believes\;D\overset{SK}{↔}MD$$

4. Derivations

Based on the idealizations, the assumptions, the BAN-logic rules, and the intermediate results of the derivations, the goals set are deduced.

From (I1):

(***D*1**)  $$\;GCS\;sees\;\langle ID_{MISSION},\;g^{x},\;ts_{1}\rangle {}_{p}$$
(***D*2**)  $$\;GCS\;believes\;D\;said\;\left[ID_{MISSION},\;g^{x},\;ts_{1}\right]\;by\;\left(D1\right),\;\left(A1\right),\;MM$$
(***D*3**)  $$\;GCS\;believes\;D\;believes\;\left[ID_{MISSION},\;g^{x},\;ts_{1}\right]\;by\;\left(D2\right),\;\left(A2\right),\;FR,\;NV$$
(***D*4**)  $$\;GCS\;believes\;D\;said\;ID_{MISSION}\;by\;\left(D3\right),\;BC$$
(***D*5**)  $$\;GCS\;believesGCS\overset{g^{XY}}{↔}D\;by\;\left(D2\right),BC,\left(A3\right),DH$$
(***D*6**)  $$\;GCS\;believesGCS\overset{AK}{↔}D\;by\;\left(D5\right),\left(A2\right),BC$$
(***D*7**)  $$\;GCS\;believesGCS\overset{EK}{↔}D\;by\;\left(D5\right),\;\left(A2\right),BC$$

From (I2):

(***D*8**)  $$\;D\;sees\langle ID_{MISSION},\;g^{y},\;ts_{2},CMD,GCS\overset{AK}{↔}D,GCS\overset{EK}{↔}D\rangle_{AK},\;\langle g^{y},\;ts_{2}\rangle {}_{PU{\left(GCS\right)}^{-1}}$$
(***D*9**)  $$\;D\;believes\;GCS\;said\;\left[g^{y},\;ts_{2}\right]\;by\;\left(D8\right),BC,\left(A4\right),MM$$
(***D*10**)  $$\;D\;believes\;GCS\;believes\;\left[g^{y},\;ts_{2}\right]\;by\;\left(D9\right),\left(A5\right),FR,NV$$
(***D*11**)  $$\;D\;believesGCS\overset{g^{XY}}{↔}D\;by\;\left(D9\right),BC,\left(A6\right),DH$$
(***D*12**)  $$\;D\;believes\;GCS\overset{AK}{↔}D\;by\;\left(D11\right),\;\left(A7\right),BC$$
(***D*13**)  $$\;D\;believes\;GCS\overset{EK}{↔}D\;by\;\left(D11\right),\left(A7\right),BC$$
(***D*14**)  $$\;D\;sees\;\langle ID_{MISSION},\;g^{y},\;ts_{2},CMD,GCS\overset{AK}{↔}D,GCS\overset{EK}{↔}D\rangle_{AK}\;by\;\left(D10\right),DR$$
(***D*15**)  $$\;D\;believes\;GCS\;said\;\left[ID_{MISSION},\;g^{y},\;ts_{2},CMD,GCS\overset{AK}{↔}D,GCS\overset{EK}{↔}D\right]\;by\;\left(D14\right),\left(D12\right),MM$$
(***D*16**)  $$\;D\;believes\;GCS\;believes\;\left[ID_{MISSION},\;g^{y},\;ts_{2},CMD,GCS\overset{AK}{↔}D,GCS\overset{EK}{↔}D\right]\;by\;\left(D15\right),\left(A5\right),FR,NV$$
(***D*17**)  $$\;D\;believes\;GCS\;believes\;ID_{MISSION}\;by\;\left(D16\right),\;BC$$
(***D*18**)  $$\;D\;believes\;GCS\;believes\;CMD\;by\;\left(D16\right),\;BC$$
(***D*19**)  $$\;D\;believes\;GCS\;believes\;GCS\overset{AK}{↔}D\;by\;\left(D16\right),\;BC$$
(***D*20**)  $$\;D\;believes\;GCS\;believes\;GCS\overset{EK}{↔}D\;by\;\left(D16\right),\;BC$$

From (I3):

(***D*21**)  $$\;GCS\;sees\;\langle ID_{MISSION},ID_{D},ID_{GCS},\;ts_{3},\;CMD,\;GCS\overset{AK}{↔}D,GCS\overset{EK}{↔}D\rangle_{AK}$$
(***D*22**)  $$\;GCS\;believes\;D\;said\;\left[ID_{MISSION},ID_{D},ID_{GCS},\;ts_{3},\;CMD,\;GCS\overset{AK}{↔}D,GCS\overset{EK}{↔}D\right]\;by\;\left(D21\right),\left(D6\right),MM$$
(***D*23**)  $$\;GCS\;believes\;D\;believes\;\left[ID_{MISSION},ID_{D},ID_{GCS},\;ts_{3},\;CMD,\;GCS\overset{AK}{↔}D,GCS\overset{EK}{↔}D\right]\;by\;\left(D22\right),\left(A8\right),NV,FR$$
(***D*24**)  $$\;GCS\;believes\;D\;believes\;ID_{D}\;by\;\left(D23\right),BC$$
(***D*25**)  $$\;GCS\;believes\;D\;believes\;CMD\;by\;\left(D23\right),BC$$
(***D*26**)  $$\;GCS\;believes\;D\;believes\;GCS\overset{AK}{↔}D\;by\;\left(D23\right),BC$$
(***D*27**)  $$\;GCS\;believes\;D\;believes\;GCS\overset{EK}{↔}D\;by\;\left(D23\right),BC$$

From (I4):

(***D*28**)  $$\;D\;sees\;\langle ID_{MISSION},\;ID_{GCS},\;ID_{D},\;ts_{4},D\overset{SK}{↔}MD,CMD\rangle_{AK}$$
(***D*29**)  $$\;D\;believes\;GCS\;said\;\left[ID_{MISSION},ID_{GCS},ID_{D},\;ts_{4},D\overset{SK}{↔}MD,\;CMD\right]by\;\left(D28\right),\left(D12\right),MM$$
(***D*30**)  $$\;D\;believes\;GCS\;believes\;\left[ID_{MISSION},ID_{GCS},ID_{D},\;ts_{4},D\overset{SK}{↔}MD,CMD\right]\;by\;\left(D29\right),\left(A9\right),FR,\;NV$$
(***D*31**)  $$\;D\;believes\;GCS\;believes\;ID_{GCS}\;by\;\left(D30\right),BC$$
(***D*32**)  $$\;D\;believes\;GCS\;believes\;D\overset{SK}{↔}MD\;by\;\left(D30\right),BC$$
(***D*33**)  $$\;D\;believes\;D\overset{SK}{↔}MD\;by\;\left(D32\right),\left(A9\right),JR$$

From the above analysis, it is shown that the SP-D2GCS protocol fulfills each of the goals (G1~G16). Moreover, the following lemmas can be derived while showing that the target security requirements are satisfied.

**Lemma** **1.** *The SP-D2GCS protocol provides a mutual authentication between D and GCS.*

**Proof.**  Through the beliefs (D4) and (D17), both D and GCS can believe ID_MISSION_. Also, they can believe ID of another from derived beliefs (D24) and (D31). Accordingly, this proves that D and GCS mutually authenticate each other. □

**Lemma** **2.** *The SP-D2GCS protocol enables a secure exchange of AK and EK keys between D and GCS.*

**Proof.**  As shown in the derivations (D5) and (D11), both GCS and D believe the session key (g^XY^) is a secret key shared between them and only known to them. There are direct beliefs that AK and EK are securely exchanged between GCS and D, as shown in (D6) and (D7) and (D12) and (D13). Also, indirect beliefs of GCS and D are shown in (D19) and (D20) and (D26) and (D27). Accordingly, it can prove that D and GCS securely exchange AK and EK. □

**Lemma** **3.** *The SP-D2GCS protocol enables a secure exchange of SK key between D and GCS.*

**Proof.**  The session key SK, which is used for communication between D and MD, is generated by GCS. According to (D32) and (D33), D believes SK as a secret key between itself and MD. Note that we cannot reason about the MD’s belief on SK because it is not involved in this protocol. However, the above-obtained belief can be evolved to allow MD to be sure of SK with the help of ST(D) during the SP-D2MD protocol. Therefore, we can prove that SK is securely exchanged between D and MD. □

**Lemma** **4.** *The SP-D2GCS protocol has resistance against denial-of-service attacks.*

**Proof.**  (D3) shows that GCS authenticates message and its freshness prior to the expensive computations, thus protecting the protocol from resource exhaustion attacks. □

**Lemma** **5.** *The SP-D2GCS protocol supports non-repudiation.*

**Proof.**  Every message of the SP-D2GCS protocol contains the public key encryption. Thus, the message can prove who transferred messages with the public key. □

**Lemma** **6.** *The SP-D2GCS protocol supports confidentiality of CMD.*

**Proof.**  In the case of GCS, (D18) and (D25) can verify that D believes the operation command CMD. Besides, D can verify that GCS sends the operation command CMD as it is encrypted by EK (which is generated by the session key g^XY^ that both D and GCS believe). Thus, D and GCS support confidentiality for operational command CMD. □

**Lemma** **7.** *The SP-D2GCS protocol supports the integrity and data authentication of messages.*

**Proof.**  Concerning GCS, (D3) and (D23) show that D verifies (I1) and (I3), which illustrates the integrity and data authentication of the message. In the case of D, (D10) and (D30) show that the GCS confirms the trust of (I2) and (I4) (respectively) to support the integrity and data authentication of the message. Accordingly, it can be shown that SP-D2GC supports integrity and data authentication for messages. □

**Lemma** **8.** *The SP-D2GCS protocol prevents the man-in-the-middle attacks.*

**Proof.**  The ECDHE public keys exchanged between D and MD are protected by the digital signatures that are also sent along with the keys. Also, it can be confirmed from (D5) and (D11) that both parties can trust the ECDHE public key. Accordingly, the SP-D2GCS protocol is secure against man-in-the-middle attacks. □

**Lemma** **9.** *The SP-D2GCS protocol supports PFS and PBS.*

**Proof.**  Lemmas 2 and 8, above, show that g^XY^ is securely set up between D and GCS. The private keys X and Y are immediately removed from both parties so that g^XY^ will not be recovered in any case. Accordingly, it can be seen that the AK and EK derived from g^XY^ support PFS and PBS. □

Hence, it can be concluded from the proofs that the SP-D2GCS protocol fulfills the security requirements outlined in Section 3, which enables it to withstand known attacks.

#### 4.1.2. SP-D2MD

1. Idealization

The idealized forms of the SP-D2MD protocol are shown below:

(**I1**)  $$\;D\to MD:\langle ID_{MISSION},ID_{MD},g^{x},\;ts_{1},D\overset{SK}{↔}MD\rangle_{SK}$$
(**I2**)  $$\;MD\to D:\;\langle ID_{MISSION},ID_{MD},g^{y},\;ts_{2},D\overset{AK}{↔}MD,D\overset{EK}{↔}MD\rangle_{AK},\;\langle g^{y},\;ts_{2},D\overset{SK}{↔}MD\rangle_{SK}$$
(**I3**)  $$\;D\to MD:\;\langle ID_{MISSION},ID_{D},ID_{MD},g^{y},\;ts_{3},D\overset{AK}{↔}MD,D\overset{EK}{↔}MD\rangle_{AK}$$

2. Assumptions

The following are the assumptions considered while preparing the derivation process. The assumptions (A1)~(A6) are related to MD and the rest are related to D.

(**A1**)  $$\;MD\;believes\;D\overset{SK}{↔}MD$$
(**A2**)  $$\;MD\;believes\;fresh\left(ts_{1}\right)$$
(**A3**)  $$\;MD\;believes\;\overset{g^{Y}}{\to }MD$$
(**A4**)  $$\;D\;believes\;D\overset{SK}{↔}MD$$
(**A5**)  $$\;D\;believes\;fresh\left(ts_{2}\right)$$
(**A6**)  $$\;D\;believes\;\overset{g^{X}}{\to }D$$
(**A7**)  $$\;MD\;believes\;fresh\left(ts_{3}\right)$$

3. Goals

The goals that are expected to be achieved by SP-D2MD are shown below:

(***G*1**)  $$\;MD\;believes\;D\;believes\;D\overset{SK}{↔}MD$$
(***G*2**)  $$\;MD\;believes\;D\overset{AK}{↔}MD$$
(***G*3**)  $$\;MD\;believes\;D\overset{EK}{↔}MD$$
(***G*4**)  $$\;D\;believes\;MD\;believes\;D\overset{SK}{↔}MD$$
(***G*5**)  $$\;MD\;believes\;D\overset{AK}{↔}MD$$
(***G*6**)  $$\;MD\;believes\;D\overset{EK}{↔}MD$$
(***G*7**)  $$\;D\;believes\;MD\;believes\;ID_{MISSION}$$
(***G*8**)  $$\;D\;believes\;MD\;believes\;ID_{MD}$$
(***G*9**)  $$\;D\;believes\;MD\;believes\;D\overset{AK}{↔}MD$$
(***G*10**)  $$\;D\;believes\;MD\;believes\;D\overset{EK}{↔}MD$$
(***G*11**)  $$\;MD\;believes\;D\;believes\;ID_{MISSION}$$
(***G*12**)  $$\;MD\;believes\;D\;believes\;ID_{D}$$
(***G*13**)  $$\;MD\;believes\;D\;believes\;D\overset{AK}{↔}MD$$
(***G*14**)  $$\;MD\;believes\;D\;believes\;D\overset{EK}{↔}MD$$

4. Derivations

The following derivations show the steps taken to realize the goals:

From (I1):

(***D*1**)  $$\;MD\;sees\;\left[ST\left(D\right),\langle M_{1},\;D\overset{\;SK\;}{↔}MD,D\overset{\;SK\;}{\Leftrightarrow }MD\rangle {\;}_{SK}\right]\;by\;\left(I1\right)$$
(***D*2**)  $$\;MD\;sees\;ST\left(D\right)\;by\;\left(D1\right),\;DR$$
(***D*3**)  $$\;MD\;believes\;GCS\;believes\;\left[ID_{MISSION},ID_{MD},\;ID_{GCS},\;D\overset{\;SK\;}{↔}MD,D\overset{\;SK\;}{\Leftrightarrow }MD,\;LT\right]\;by\;\left(D2\right),\;\left(A1\right),\;MM,\;\left(A2\right),\;FR,NV$$
(***D*4**)  $$\;MD\;believes\;GCS\;believes\;D\overset{\;SK\;}{↔}MD\;by\;\left(D3\right),\;BC$$
(***D*5**)  $$\;MD\;believes\;GCS\;believes\;D\overset{\;SK\;}{\Leftrightarrow }MD\;by\;\left(D3\right),\;BC$$
(***D*6**)  $$\;MD\;believes\;D\overset{\;SK\;}{↔}MD\;by\;\left(D4\right),\;\left(A3\right),\;JR$$
(***D*7**)  $$\;MD\;believes\;D\overset{\;SK\;}{\Leftrightarrow }MD\;by\;\left(D5\right),\;\left(A4\right),\;JR$$
(***D*8**)  $$\;MD\;sees\langle M_{1},\;D\overset{\;SK\;}{↔}MD,D\overset{\;SK\;}{\Leftrightarrow }MD\rangle {\;}_{SK}by\;\left(D1\right),\;DR$$
(***D*9**)  $$\;MD\;believes\;D\;said\;\left[M_{1},\;D\overset{\;SK\;}{↔}MD,D\overset{\;SK\;}{\Leftrightarrow }MD\right]by\;\left(D8\right),\;\left(D7\right),\;MM$$
(***D*10**)  $$\;MD\;believes\;D\;believes\;\left[M_{1},\;D\overset{\;SK\;}{↔}MD,D\overset{\;SK\;}{\Leftrightarrow }MD\right]by\;\left(D9\right),\;\left(A5\right),\;FR,\;NV$$
(***D*11**)  $$\;MD\;believes\;D\;believes\;D\overset{\;SK\;}{↔}MDby\;\left(D10\right),\;BC$$
(***D*12**)  $$\;MD\;believes\;D\;believes\;D\overset{\;SK\;}{\Leftrightarrow }MDby\;\left(D10\right),\;BC$$
(***D*13**)  $$\;MD\;believes\;D\overset{\;g^{XY}\;}{↔}MD\;by\;\left(D9\right),\;BC,\;\left(A6\right),\;DH$$
(***D*14**)  $$\;MD\;believes\;D\overset{\;g^{XY\;}}{\Leftrightarrow }MD\;by\;\left(D9\right),\;BC,\;\left(A6\right),\;DH$$

From (I2):

(***D*15**)  $$\;D\;sees\;\langle M_{2},\;\langle M_{2},D\overset{\;g^{XY}\;}{↔}MD,\;D\overset{\;g^{XY\;}}{\Leftrightarrow }MD\rangle_{g^{XY}}\rangle {}_{SK}\;by\;\left(I2\right)$$
(***D*16**)  $$\;D\;believes\;MD\;said\;\left[\langle M_{2},D\overset{\;g^{XY}\;}{↔}MD,\;D\overset{\;\rangle g^{XY\;}}{\Leftrightarrow }MD_{g^{XY}}\right]\;by\;\left(D15\right),\left(A7\right),\;MM$$
(***D*17**)  $$\;D\;believes\;MD\;believes\;\left[\langle M_{2},D\overset{\;g^{XY}\;}{↔}MD,\;D\overset{\;g^{XY\;}}{\Leftrightarrow }MD\rangle_{g^{XY}}\right]\;by\;\left(D16\right),\;\left(A8\right),FR,NV$$
(***D*18**)  $$\;D\;believes\;D\overset{\;g^{XY}\;}{↔}MD\;by\;\left(D16\right),\;BC,\;\left(A9\right),\;DH$$
(***D*19**)  $$\;D\;believes\;D\overset{\;g^{XY\;}}{\Leftrightarrow }MD\;by\;\left(D16\right),\;BC,\;\left(A9\right),\;DH$$
(***D*20**)  $$\left(D20\right)\;D\;sees\langle M_{2},D\overset{\;g^{XY}\;}{↔}MD,\;D\overset{\;g^{XY\;}}{\Leftrightarrow }MD\rangle_{g^{XY}}by\;\left(D16\right),\;BC$$
(***D*21**)  $$\;D\;believes\;MD\;believes\;\left[M_{2},D\overset{\;g^{XY}\;}{↔}MD,\;D\overset{\;g^{XY\;}}{\Leftrightarrow }MD\right]\;by\;\left(D20\right),\;\left(D19\right),\;MM,\;\left(A8\right),\;FR,\;NV$$
(***D*22**)  $$\;D\;believes\;MD\;believes\;D\overset{\;g^{XY}\;}{↔}MD\;by\;\left(D21\right),\;BC$$
(***D*23**)  $$\;D\;believes\;MD\;believes\;D\overset{\;g^{XY\;}}{\Leftrightarrow }MD\;by\;\left(D21\right),BC$$

From (I3):

(***D*24**)  $$\;MD\;sees\;\langle M_{3},D\overset{\;g^{XY}\;}{↔}MD,\;D\overset{\;g^{XY\;}}{\Leftrightarrow }MD\langle_{g^{XY}}\;by\;\left(I3\right)$$
(***D*25**)  $$\;MD\;believes\;D\;believes\;\left[M_{3},D\overset{\;g^{XY}\;}{↔}MD,\;D\overset{\;g^{XY\;}}{\Leftrightarrow }MD\right]\;by\;\left(D24\right),\left(D14\right),\;MM,\left(A10\right),\;FR,\;NV$$
(***D*26**)  $$\;MD\;believes\;D\;believes\;D\overset{\;g^{XY}\;}{↔}MD\;by\;\left(D25\right),\;BC$$
(***D*27**)  $$\;MD\;believes\;D\;believes\;D\overset{\;g^{XY\;}}{\Leftrightarrow }MD\;by\;\left(D25\right),\;BC$$

From the above analysis, it is shown that the SP-D2MD protocol satisfied the goals (G1~G14). Also, the following lemmas can be derived through the satisfied requirements.

**Lemma** **10.** *The SP-D2MD protocol provides mutual authentication between D and MD.*

**Proof.**  The derivation result (D10) shows that the MD authenticates D. Similarly, D authenticates MD, as shown in (D17). Hence, mutual authentication between D and MD is realized in the SP-D2GC protocol. □

**Lemma** **11.** *The SP-D2MD protocol provides a secure key exchange of AK and EK.*

**Proof.**  As shown in the derivations (D13) and (D14) and (D18) and (D19), both MD and D believe that the session key (g^XY^) is a secret key shared between them and also believe that it is a shared secret that is only known to them. Accordingly, there is a direct belief that AK and EK are securely exchanged between GCS and D, as these keys are computed from the session key g^XY^. Also, the indirect belief was secured by trusting beliefs in AK and EK through (D22), (D23), (D26), and (D27). Thus, AK and EK are exchanged securely between D and MD. □

**Lemma** **12.** *The SP-D2MD protocol prevents denial-of-service attacks.*

**Proof.**  In the case of MD, M1 shows freshness through (D10) and does not issue a message without knowing SK, thus supporting defense against denial-of-service attacks. In the case of D, M2 is protected by AK, which is derived from the master session key (g^XY^). As a result, the next message will not be processed by MD since the sender has no knowledge of the master session key; thus, supporting denial-of-service attacks. □

**Lemma** **13.** *The SP-D2MD protocol supports confidentiality of AK and EK.*

**Proof.**  In the case of MD, (D13) and (D14) show the secure exchange of AK and EK, which indicates the confidentiality of AK and EK. Similarly, D can be sure about the confidentiality of AK and EK, as shown in (D18) and (D19). □

**Lemma** **14.** *The SP-D2MD protocol supports confidentiality of SK.*

**Proof.**  The proof for Lemma 3 of the SP-D2GCS protocol shows that SK is exchanged between D (MD) and GCS securely. The proof of Lemma 8 shows the confidentiality of SK between D and GCS. Similarly, it can be shown that the SP-D2MD protocol supports the confidentiality of SK, as indicated in the derivations (D6) and (D7). □

**Lemma** **15.** *The SP-D2MD protocol supports integrity and data authentication of messages.*

**Proof.**  The derivations (D10) and (D25) show that D supports the integrity and data authentication of the message by verifying the trust of M1 and M3. MD also verifies the trust of M2, through the derivation (D17), to support the integrity and data authentication of the message. Hence, we can verify that D and MD support the integrity and data authentication of the message. □

**Lemma** **16.** *The SP-D2MD protocol provides defense against man-in-the-middle attacks.*

**Proof.**  The ECDHE public keys exchanged between D and MD are protected by the digital signatures that are also sent along with the keys. Also, it can be confirmed from (D10) and (D17) that both parties can trust the ECDHE public key. Accordingly, the SP-D2MD protocol is secure against man-in-the-middle-attack. □

**Lemma** **17.** *The SP-D2MD protocol supports PFS and PBS.*

**Proof.**  As per Lemma 11 and Lemma 12 of the SP-D2MD protocol, the master session key g^XY^ is securely set up through the Diffie–Hellman key exchange between M and MD. The private keys X and Y are immediately removed from both parties so that g^XY^ is not recovered under any circumstances. Hence, the authentication and encryption keys derived from g^XY^ support PFS and PBS. □

From the above proofs, we can conclude that SP-D2MD, like SP-D2GCS, is proven to satisfy mutual authentication, secure key exchange, integrity and data authentication of messages, and supports PFS, which makes it secured against known attacks.

### 4.2. Formal Verification with Scyther

Although the formal verification carried out by BAN-Logic validates the proposed protocol, highlighting that it meets the security goals and is secure against known attacks, BAN-Logic has found to have a limitation in pointing out some flaws [34]. Hence, for a complete formal analysis of security protocols, it is often necessary to combine BAN-Logic with automated tools such as Scyther and AVISPA (Automated Validation of Internet Security Protocols and Applications) [35]. In this paper, the automated formal verification tool Scyther is used to formally verify the SP-D2GC and SP-D2MD protocols.

Scyther, developed by Cremers in 2007, provides a graphical user interface that integrates the Command Line tool and the python scripting interface as an automated tool for formal validation. It provides validation, presentation, analysis, specification, and derivation of protocols. In particular, by providing protocol behavior classes, Scyther points out security problems through straightforward formalization and verification of protocols. The Security Protocol Description Language (SPDL) used in Scyther has a similar syntax to C/JAVA language (although case-insensitive), and defines roles as a series of events, consisting of events representing transmission and reception of information.

For protocol verification, Scyther can be used in three ways. Verification claim: verified or falsified security attributes, automatic claims: Scyther automatically generates and confirms a claim when security attributes are not specified as a claim event, and characterization: Scyther analyzes protocols and provides a finite representation of all traces, including the execution of protocol roles, so that each protocol role can be characterized. During the protocol verification process, Scyther creates an attack graph for unsafe protocols, and displays an individual attack graph for each claim. Claim events used for verification in this paper can be categorized by the functions shown in Table 4, and the details are described in Reference [26].

**Figure 6:**
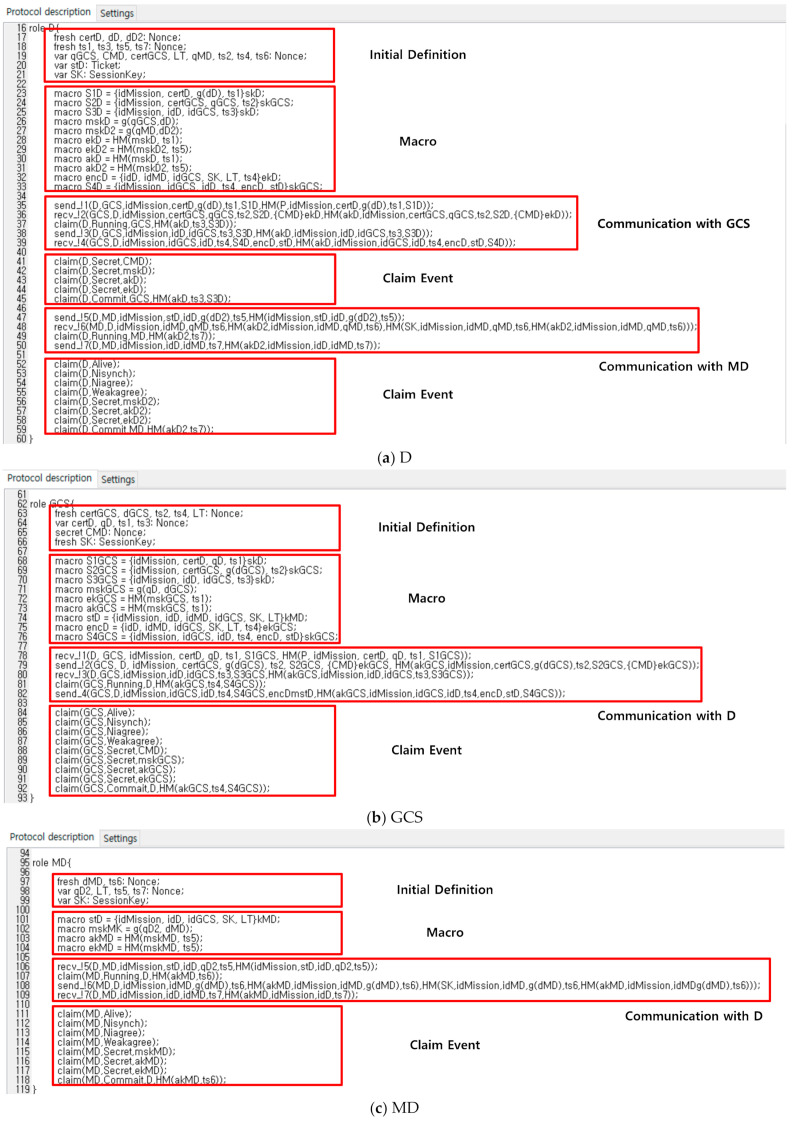
SPDL script of proposed protocol; (**a**) D’s SPDL script; (**b**) GCS’s SPDL script; (**c**) MD’s SPDL script.

At first, each role is modeled in SPDL scripts. The basic roles include the D’s role, the GCS’s role, and the MD’s role, as shown in Figure 6a–c, respectively. In addition, we included the claim events to each modeling, such as Alive, Nisynch, Niagree, Weakagree, Commit/Running, and Secret. Each roles are communicated with each other through the channel set through ‘send’ and ‘recv’. These events check whether modeling can provide authentication and secrecy. If the proposed protocol is secure, the status of the result will show that every claim is OK. Otherwise, the result will show the process of leading to a vulnerable modeling state.

**Figure 7:**
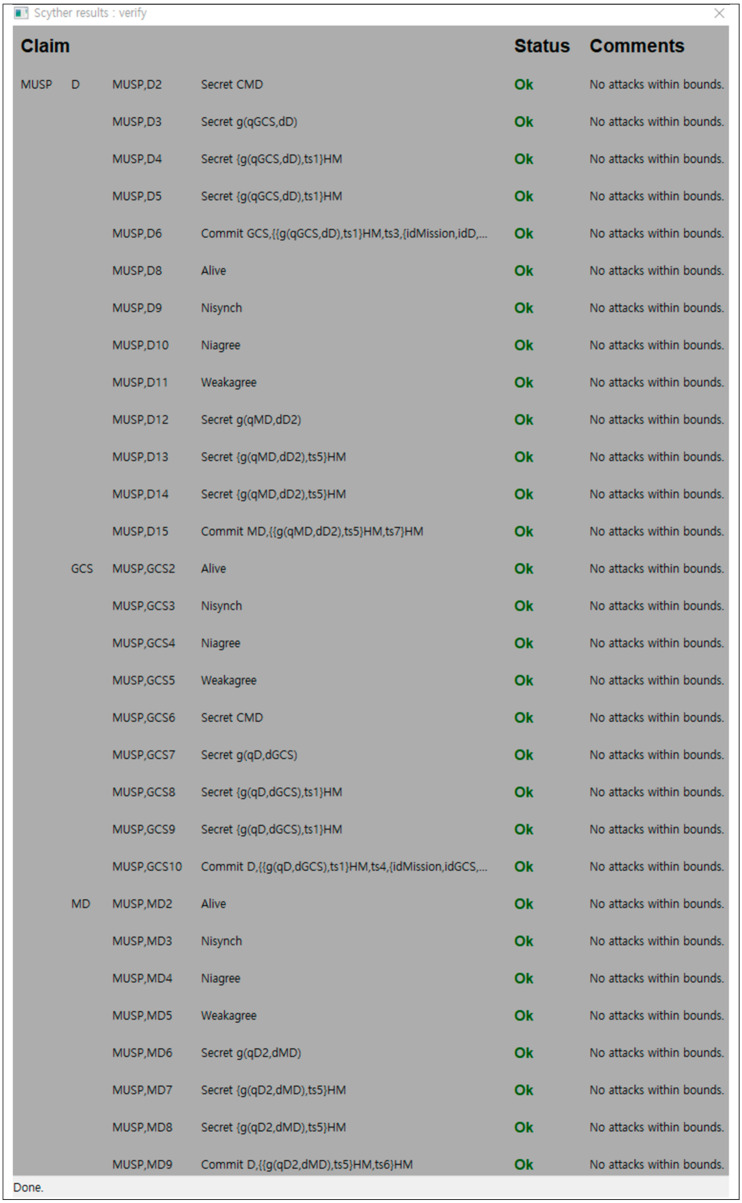
A Scyther verification result.

Scyther composes a communication environment based on SPDL scripts, as shown in Figure 6, and executes verification according to claim events. As shown in Figure 7, D, GCS, and MD of the proposed protocol have not been attacked against claim events such as Alive, Nisynch, Niagree, Weakagree, Commit/Running, and Secret. Consequently, the proposed protocol is proven to be secure against known attacks.

## 5. Performance Analysis

In this section, the proposed protocol is compared with four state-of-the-art security protocols [18,23,27,36], that can be deployed to protect the communication within the UAV network. The comparison is made in terms of security and computation overhead, whose results are provided in Table 5 and Table 6, respectively.

Table 5 provides the comparative analysis among protocols based on the security properties. It can see that the work in References [23,27] does not support non-repudiation property. Also, References [18,27] do not provide PFS. Therefore, if any long-term key used to derive past session keys has been exposed, adversaries can use the session keys to recover the encrypted messages to acquire sensitive data. Likewise, References [18,27] do not support PBS, thus causing the subsequent sessions to be vulnerable to various attacks, in case of compromise of any of the current long-term keys. Moreover, proposed protocols of References [23,36] are susceptible to DoS attacks due to resource exhaustion. Even worse, they perform high computational operation in order to support PFS and PBS, which puts a heavy burden on key updates during flight. In addition, protocols in References [18,23,36] do not support security between UAVs. As a result, it can be concluded that the designed security protocol offers better security compared to the other state-of-the-art protocols.

On the other hand, Table 6 compares the proposed protocol with the 4 protocols based on computation overhead. Similar to References [18,27], the proposed protocol cannot avoid excessive computational overhead in SP-D2GCS to support PFS and PBS. It is worth to note that such overhead is negligible because SP-D2GCS is executed only once. However, based on the strong session key, SK, derived from SP-D2GCS, SP-D2MD, which is primary executed in the proposed protocols, achieves relatively lightweight computation while meeting the security requirements.

## 6. Simulation Results

**Figure 8:**
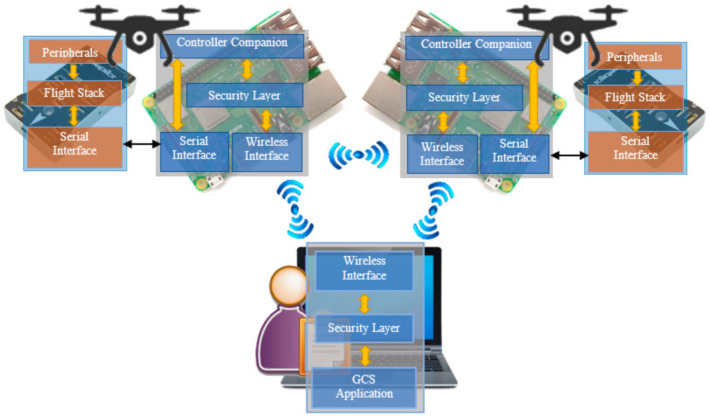
An illustration of UAV ad-hoc network architecture implemented in the experimental simulation.

**Figure 9:**
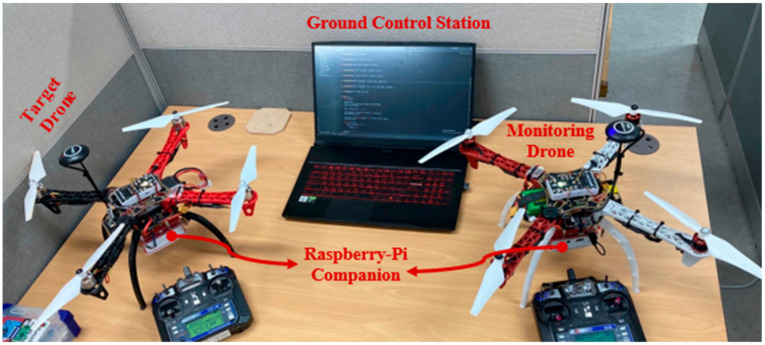
The actual experimental testbed for the proposed security protocol.

We developed the proposed security protocols using Python and tested it on an ad-hoc network that composed two real UAVs and a ground control station. The network architecture in the experimental simulation along with the actual experimental test bed for the proposed protocol are shown in Figure 8 and Figure 9, respectively. The instruments used in this experiment are also listed in Table 7. The UAVs are equipped with a companion board Raspberry-Pi that is serially interfaced with the Pixhawk flight controller. The companion board enables developers to develop a self-operating UAV according to their target application. In the experiments, we create a straightforward application where UAVs and GCS simply exchange operational data or commands with each other at a pre-defined interval. Meanwhile, before the execution of said application, the proposed security protocols were first accomplished. During the execution of the protocols, essential metrics, such as size and transmission latency of the messages, were collected. The transmission latency refers to the amount of time for a message to travel across the network.

Table 8 shows the collected values of the target metrics. Based on this, the proposed D2GCS and D2MD security protocols have a total message size of 2411 and 781 bytes, respectively. Furthermore, the average transmission latency of each message corresponds to the number of bytes it carries. Based on our experiment, it takes approximately 213 milliseconds to establish a secure channel between UAV and GCS. Meanwhile, the execution of the D2MD security protocol takes an average of 29 milliseconds. The performance of UAVs can be significantly influenced by its power consumption and transmission latency, which can be associated to the message size of a particular key exchange protocol. With regards to the former, the size of the transmitted or received messages play an important role in extending energy lifetime of UAVs, especially when the key exchange protocol is executed during its flight. On the other hand, the latter, which is still dependent on the size of the messages, has an impact on the amount of time it takes for two parties to establish the secure channel. In relation to these factors, the relatively low message size and latency obtained from our experiment indicate that the proposed protocol has a great potential in terms of the practical aspects related to UAV network security.

## 7. Conclusions

Although UAVs play an essential role in a wide range of application areas, there are still security issues that limit their full potential in delivering the required solution. Especially in the case of military scenarios, the security and privacy of UAVs should be among the highest priority. In order to resolve the security concerns, we proposed a security protocol (with two sub-protocols, SP-D2GCS and SP-D2MD) that enables secure communication among UAVs and between the UAV and the GCS.

Our protocol can be applied in four different deployment scenarios. Scenario one consists of multiple military UAVs with inbuilt sensors that transmit traffic to each other, in which only the monitoring drone is able to communicate with GCS directly. In this case, the SP-D2GCS protocol assists the communication between the drone and GCS, while SP-D2MD is used between the drone and monitoring drone. In case 2, apart from the communication between the drones and monitoring drones, the ordinary drones themselves communicate with each other. However, similar to case 1, it is only the monitoring drone that communicates with the GCS. The third case involves direct communication between the drones and the GCS without a monitoring node sitting between them. In such case, the SP-D2GCS protocol can be used to secure the channel. The final arrangement is similar to case two, except all intercommunicating drones also communicate with the GCS directly, which uses both of the proposed sub-protocols.

Our protocol is also evaluated to prove that it meets all the security requirements described in the proposed protocol section. The proof is conducted by using two formal verification methods, BAN-Logic and Scyther. Furthermore, both sub-protocols are implemented on a real UAV (powered by Raspberry Pi) and a Linux-based ground control station and compared to other similar protocols against security and performance. The authors would like to further consider the privacy issues in UAV communication and design an adaptive security solution as their future work.

## Figures and Tables

**Table 1:** Notations and their meaning.

Notation	Description
D	Drone.
MD	Monitoring Drone.
GCS	Ground Control Station.
ECDH	Elliptic Curve Diffie–Hellman.
ECDSA	Elliptic Curve Digital Signature Algorithm.
HMAC	Hash-based Message Authentication Code
ID_MISSION_	Operation ID.
P	PIN number.
d_X_	X’s ECDH Private key.
Q_X_	X’s ECDH Public key: d_X_ • G.
PU(X)	X’s ECDSA Public key.
PR(X)	X’s ECDSA Private key.
HM(K, M)	An HMAC function where K is a secret and M is an input message.
CERT_X_	X’s Digital Certificate.
ts	Timestamp.
CMD	Operation command.
SK	Session key.
MSK_X-Y_	Master session key shared between X and Y.
EK_X-Y_	Encryption key shared between X and Y.
AK_X-Y_	Authentication key shared between X and Y.
ST(X)	X’s Authentication Ticket.
LT	Key life cycle (Lifetime).
E(K, M)	An encrypt function where K is a secret key and M is an input message.
D(K, C)	A decrypt function where K is a secret key and C is a cipher message.

**Table 2:** BAN-Logic Notations.

Notations	Meanings
P believes X	P believes that the message X is true
P sees X	P receives the message X at any point in time
P said X	P previously sent the message X
P controls X	P has jurisdiction over X
$Fresh\;\left(X\right)\;$	X is fresh
$P\overset{K}{↔}Q\;$	K is a secret key shared between P and Q
$\overset{K}{\to }P\;$	K is the P’s public key and L is the P’s private key
$P\overset{K}{\Leftrightarrow }Q\;$	K is a shared secret between P and Q
${\left\{X\right\}}_{K}\;$	X is encrypted with a key K
X, Y	X is combined with Y

**Table 3:** BAN-Logic Rules.

Rule Names	Rules
Message Meaning Rule (MM)	$\frac{P\;believes\;P\overset{K}{↔}Q,\;P\;sees\;{\left\{X\right\}}_{K}\;}{P\;believes\;Q\;said\;X}\;\frac{P\;believes\;P\overset{K}{\Leftrightarrow }Q,\;P\;sees\;X_{K}\;}{P\;believes\;Q\;said\;X}\;\frac{P\;believes\;\overset{K}{\to }Q,\;P\;sees\;{\left\{X\right\}}_{L^{-1}}\;}{P\;believes\;Q\;said\;X}$
Nonce Verification Rule (NV)	$\frac{P\;believes\;\#\left(X\right),\;P\;believes\;Q\;said\;X}{P\;believes\;Q\;believes\;X}$
Jurisdiction Rule (JR)	$\frac{P\;believes\;Q\;controls\;X,\;P\;believes\;Q\;believesX}{P\;believesX}$
Freshness Rule (FR)	$\frac{P\;believes\;fresh\left(X\right)}{P\;believesfresh\left(X,Y\right)}$
Decomposition Rule (DR)	$\frac{P\;sees\;\left(X,\;Y\right)}{P\;sees\;X}$
Belief Conjunction Rule (BC)	$\frac{P\;believes\;X,\;P\;believes\;Y\;}{P\;believes\left(X,Y\right)}\;\frac{P\;believes\;Q\;believes\;\left(X,Y\right)\;}{P\;believes\;Q\;believes\;X}\;\frac{P\;believes\;Q\;said\;\left(X,Y\right)\;}{P\;believes\;Q\;said\;X}$
Diffie–Hellman Rule (DH)	$\frac{P\;believes\;Q\;said\;\overset{g^{Y}}{\to }Q,\;P\;believes\;\overset{g^{X}}{\to }P\;}{P\;believes\;P\overset{g^{XY}}{↔}\;Q}\;\frac{P\;believes\;Q\;said\;\overset{g^{Y}}{\to }Q,\;P\;believes\;\overset{g^{X}}{\to }P\;}{P\;believes\;P\overset{g^{XY}}{\Leftrightarrow }\;Q}$

**Table 4:** Claim event description.

Notations	Meanings
Event	Security Attribute
Alive, Nisynch, Niagree, Weakagree, Commit	Authentication
Secret	Secrecy

**Table 5:** The state-of-the-art comparison with existing protocols.

Security Requirements	[18]	[23]	[27]	[36]	Our Protocol
Confidentiality	✓	✓	✓	✓	✓
Integrity	✓	✓	✓	✓	✓
Mutual Authentication	✓	✓	✓	✓	✓
Non-repudiation	✓	X	X	✓	✓
Perfect Forward Secrecy	X	✓	X	✓	✓
Perfect Backward Secrecy	X	✓	X	✓	✓
Response to DoS Attacks	✓	X	✓	X	✓
Man-in-the-middle response	✓	✓	✓	✓	✓
D2D security support	X	X	✓	X	✓

✓: Supported, X: Unsupported.

**Table 6:** Computational overhead comparison.

Security Protocols	Computational Overhead	
Our Protocol	SP-D2GCS	SP-D2MD
	$7C_{SC}+4C_{S}+4C_{SV}+2C_{DH}+11C_{HM}+2C_{C}$	$C_{SC}+2C_{DH}+8C_{HM}$
[18]	$5C_{WBC}+2C_{S}+2C_{SV}$	
	Initial Step	Authentication Step
[23]	-----	$2C_{SC}+3C_{XoR}+3C_{H}$
[27]	$C_{bio}+8C_{XoR}+12C_{H}$	$C_{bio}+12C_{XoR}+32C_{H}$
[36]	$2C_{PC}+2C_{S}+2C_{SV}$	

$C_{bio}$: Biometric Authentication, $C_{SC}$: Symmetric Key Cryptography, $C_{PC}$: Public Key Cryptography, $C_{DH}$: Diffie–Hellman Key Exchange, $C_{S}$: Digital Signature, $C_{sv}:$ Digital Signature Verification, $C_{WBC}$: White Box Encryption, $C_{XoR}$: XOR Operation, $C_{HM}$: HMAC operation, $C_{H}$: Hash Operation, $C_{CV}$: Digital Certificate Verification.

**Table 7:** Implementation environment.

Environment	Description
UAV	Two UAVs each with Raspberry Pi model B+
GCS	Ubuntu 18.04.3 LTS, 11GB RAM, and i5-2400 CPU @3.10 GHz
Language	Python 3.8

**Table 8:** Notations and their meaning.

Messages	SP-D2GCS		SP-D2MD	
	Message Size (bytes)	Latency (ms*)	Message Size (bytes)	Latency (ms*)
M1	939	71.11001	393	18.74995
M2	1036	93.67990	257	10.45012
M3	218	23.38982	131	9.96995
M4	218	25.03991	-	-
Total	2411	213.2196	781	29.20008

* ms: millisecond.

## Data Availability

Not applicable.
